# Prognostic value of the modified model for end-stage liver disease (MELD) score including albumin in acute heart failure

**DOI:** 10.1186/s12872-021-01941-7

**Published:** 2021-03-09

**Authors:** Shengen Liao, Xinyi Lu, Iokfai Cheang, Xu Zhu, Ting Yin, Wenming Yao, Haifeng Zhang, Xinli Li

**Affiliations:** Department of Cardiology, Jiangsu Province Hospital and Nanjing Medical University First Affiliated Hospital, Guangzhou Road 300, Nanjing, 210029 China

**Keywords:** Model for end-stage liver disease, Mortality, Albumin, Acute heart failure

## Abstract

**Background:**

Liver and renal function evaluated by the model for end-stage liver disease (MELD) score, the MELD excluding the international normalized ratio (MELD_XI) score and the MELD including sodium (MELD_sodium) score have been considered predictors of adverse events for patients with acute heart failure (AHF). However, the prognostic value of the MELD including albumin (MELD_albumin) score in patients with AHF has not been assessed.

**Methods:**

A total of 466 patients with AHF were prospectively evaluated. We compared the accuracy of the 4 MELD score formulas using the time-dependent receiver operating characteristic (ROC) curve and corresponding areas under the curve (AUC).

**Results:**

During a median follow-up period of 34 months, 196 deaths occurred. In the fully adjusted Cox regression model, standardized hazard ratios with 95% confidence interval expressing the risk of all-cause mortality were 1.22 (1.06–1.40), 1.20 (1.04–1.39), 1.23 (1.06–1.42) and 1.21 (1.05–1.41) for MELD, MELD_XI, MELD_sodium and MELD_albumin scores, respectively. The MELD_albumin score showed the best prognostic accuracy (AUC = 0.658) for the prediction of long-term all-cause mortality, followed by the MELD_sodium score (AUC = 0.590), the MELD score (AUC = 0.580), and the MELD_XI score (AUC = 0.544); the MELD_albumin score performs significantly more accurate than MELD and MELD_XI score for predicting the risk of all-cause mortality. Considering reclassification, MELD_albumin score increased the net reclassification improvement over and beyond MELD (13.1%, *P* = 0.003), MELD_XI (14.8%, *P* = 0.002), and MELD_sodium (11.9%, *P* = 0.006) scores for all-cause mortality.

**Conclusions:**

The MELD_albumin score increases risk stratification of all-cause mortality over and beyond the MELD score and the other modified MELD scores in patients with acute heart failure.

## Background

Heart failure (HF) is a common disease and is associated with considerable morbidity and mortality worldwide [1]. With the increase in HF patients, HF has recently become a serious health problem [2]. Acute heart failure (AHF) is a complex syndrome characterized by worsening heart failure symptoms. The predominant clinical profile in most patients with AHF is congestion and hypoperfusion, which can lead to organ dysfunction and increase the risk of mortality.

Liver and renal dysfunction are often complicated in patients with acute heart failure [3]. Complicated interaction between heart, kidney, and liver has been an object of interest for a long time. In that sense, simple score capable of quantitating the severity of multiorgan failure is attractive. Biomarkers that reflect liver and kidney function are often used to predict adverse clinical outcomes in patients with AHF [4, 5]. The model for end-stage liver disease (MELD) score evaluating liver and renal function was considered a reliable predictor for the risk of adverse events in AHF patients [6]. Several studies also focused on the effects of modified MELD versions, such as the MELD excluding the international normalized ratio (MELD_XI) score [3, 7], and the MELD including sodium (MELD_sodium) score [8], on the prognosis of acute heart failure. However, the prognostic value of the MELD including albumin (MELD_albumin) score, which includes the serum value of albumin rather than the international normalized ratio, in AHF patients is still unknown. Moreover, it is largely unknown whether one of the MELD scores or its 3 modifications is superior for predicting the risk of mortality.

Accordingly, we aimed to assess the prognostic role of the MELD_albumin score in patients with AHF over a long-term follow-up. In addition, we further aimed to compare the accuracy of the MELD_albumin score with other well-established objective MELD and modified MELD scores for predicting the risk of death in patients with AHF.

## Methods

### Study subjects

**Fig. 1:**
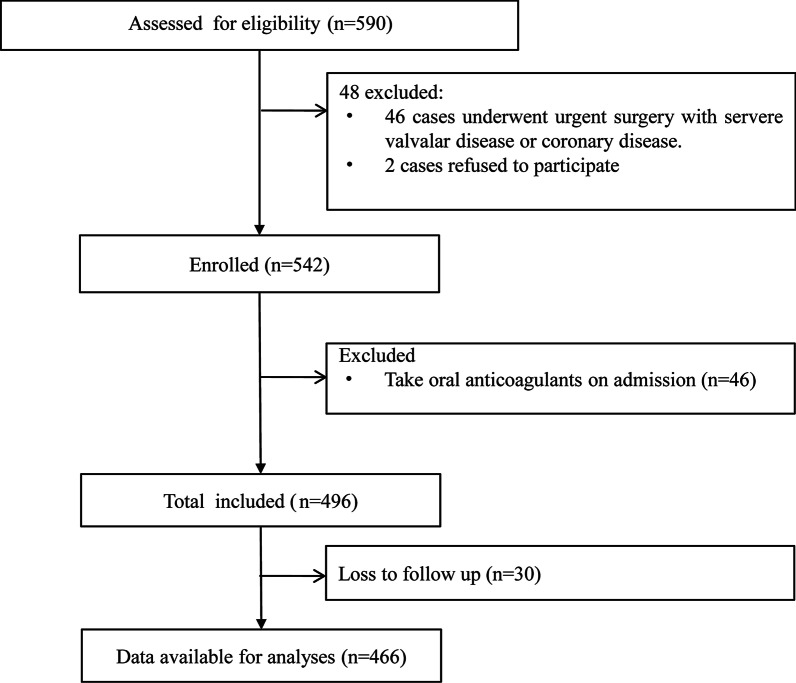
Study flow chart

We analyzed data from the registry study of AHF in China: the diagnostic standards, risk stratification, and clinical outcomes of acute heart failure (registration number: ChiCTR-ONC-12001944), which is a multicenter, observational, prospective cohort study. From March 2012 to June 2017, 590 patients with AHF were assessed for eligibility. Patients with end-stage renal dysfunction/hemodialysis and decompensated liver cirrhosis were excluded in our study. The diagnosis of AHF was based on clinical signs and symptoms and according to the guidelines, and each patient received standard clinical evaluation and guided recommended treatment [9]. Patients with severe vascular disease or coronary artery disease requiring urgent surgery (n = 46) or who withdrew informed consent (n = 2) were excluded from the study. We also excluded patients with end-stage renal disease requiring dialysis and patients who were already taking oral anticoagulants upon admission (n = 46). Finally, a total of 496 patients were included in this study (Fig. 1).

### MELD score and its 3 modifications

**Fig. 2:**
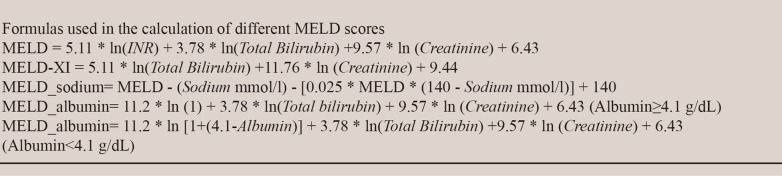
The formulas for calculating the model for end-stage liver disease (MELD) score and modified MELD scores

The standard MELD [10] score and 3 modifications (MELD_XI [11], MELD_albumin [12] and MELD_sodium [13]) were calculated according to the formulas presented in Fig. 2. If the total bilirubin or creatinine level was less than 1 mg/dl, their lower limit was assumed to be 1 mg/dl to avoid negative scores.

### Data collection

Data on age, sex, body mass index (BMI), history of diabetes mellitus, hypertension, heart rate, blood pressure on admission, New York Heart Association (NYHA) class, etiology of heart failure and prescribed medicine were recorded. Left ventricular ejection fraction (LVEF) was measured according to the modified Simpson’s method. Pulmonary hypertension (pulmonary artery systolic pressure ≥ 50 mmHg), tricuspid regurgitation severity and right ventricular diastolic diameter were estimated by transthoracic echocardiography. Blood samples to test for serum sodium, potassium, albumin, creatinine, uric acid, alanine aminotransferase (ALT), aspartate aminotransaminase (AST), total bilirubin, the international normalized ratio (INR) and pro-brain natriuretic peptide (proBNP) concentrations were obtained on admission.

### Endpoints and follow up

The primary endpoint was all-cause mortality. The secondary endpoint was cardiovascular mortality due to AHF. Cardiovascular mortality was coded according to the International Classification of Diseases, 10th Revision (ICD–10). All patients were followed-up in our cardiology department at 3, 6, and 12 months after discharge and then every 6 months thereafter.

### Statistical analysis

Categorical variables are presented as percentages. Continuous variables are presented as the mean (standard deviation, SD) or median (interquartile) according to the distribution. Characteristics were compared according to survival status using the independent Student's t-test, the Wilcoxon test and the chi-square test, as appropriate. Comparisons between MELD and its 3 modifications were performed by the Wilcoxon test. Pairwise Spearman correlation analysis was used to investigate the relationships among MELD score and its 3 modifications.

We evaluated the association between the MELD scores and each of our outcomes using Cox proportional hazards regression with 1-SD increments in the continuously distributed MELD score and its 3 modifications. Univariate Cox regression analysis for all-cause mortality indicated that variables with statistically significant differences (*P*<0.05) were used to derive the final model. The statistically significant variables were assessed in terms of the outcomes of all-cause mortality and cardiovascular mortality. For each outcome, we calculated unadjusted hazard ratios (HRs); HRs adjusted for age and sex (Model 1); and HRs adjusted for age, sex, NYHA class, LVEF, proBNP, systolic blood pressure and diastolic blood pressure (Model 2) along with corresponding 95% confidence intervals (CIs).

The predictive performance of the MELD scores was calculated according to the time-dependent receiver–operator characteristic (ROC) curves [14] and the corresponding areas under the curve (AUCs). Comparisons of the AUCs were performed with a DeLong test [15], and . Additionally, we analysed the reclassification ability of MELD and its 3 modifications using the continuous net reclassification improvement (NRI) analysis. All analyses were performed using R software version 3.6.0 (R Foundation for Statistical Computing, Vienna, Austria). Bonferroni's correction (*P* < 0.016 was considered statistically significant) was applied to adjust for multiple comparisons among MELD score and its three modifications, and *P* value < 0.05 was considered statistically significant in other statistical analysis.

## Results

### Baseline characteristics

Thirty patients were lost to follow-up. A total of 466 patients with AHF were included in the final analysis. After a median follow-up of 34 months, 196 (42.1%) deaths occurred, and 158 (33.9%) patients died of cardiovascular causes. At baseline, patients who died were older than survivors and were more often female. Notably, patients who died had more severe clinical symptoms, lower systolic or diastolic blood pressure, lower serum albumin, lower LVEF and higher proBNP (Table 1).

**Table 1:** Baseline characteristics of the enrolled patients

Variables	All (n = 466)	Deaths (n = 196)	Survivors (n = 270)	*P* value
Age (years)	61.8 (25.0)	65.0 (15.5)	59.5 (29.9)	0.019
Male, %	314 (67.4%)	111 (56.6%)	203 (75.2%)	< 0.001
Diabetes,%	118 (25.3%)	51 (26.0%)	67 (24.8%)	0.768
Hypertension, %	244 (52.4%)	97 (49.5%)	147 (54.4%)	0.290
Ischemic heart failure, %	125 (26.8%)	53 (27.0%)	72 (26.7%)	0.928
*NYHA*				0.041
II	57 (21.1%)	24 (12.2%)	81 (17.4%)	
III	138 (51.1%)	108 (55.1%)	246 (52.8%)	
IV	75 (27.8%)	64 (32.7%)	139 (29.8%)	
Heart rate, beats/min	85.5 (20.9)	85.1 (21.4)	85.7 (20.6)	0.731
Systolic blood pressure (mmHg)	127 (22.6)	124 (19.9)	129 (24.2)	0.020
Diastolic blood pressure (mmHg)	78.5 (15.2)	76.0 (12.2)	80.4 (16.8)	0.002
Potassium (mmol/L)	3.99 (0.51)	3.99 (0.51)	3.99 (0.50)	0.912
Sodium (mmol/L)	140 (3.9)	139 (4.2)	140 (3.7)	0.086
Albumin (g/L)	3.66 (0.48)	3.60 (0.49)	3.71 (0.47)	0.025
Creatinine (mg/dL)	1.16 (0.65)	1.21 (0.61)	1.12 (0.68)	0.131
Uric acid (mg/dL)	8.13 (2.83)	8.41(3.06)	7.93 (2.64)	0.070
ALT (U/L)	27(17–46)	26(15–50)	27(18–44)	0.550
AST (U/L)	28(22–42)	28(22–48)	28(22–39)	0.162
INR	1.20 (0.28)	1.22 (0.26)	1.18 (0.30)	0.142
Total bilirubin (mg/dL)	1.23 (1.03)	1.28 (1.00)	1.19 (1.04)	0.348
proBNP (ng/L)	2254 (1269–5835)	2873 (1572–7373)	1779 (1087–4601)	< 0.001
CRP (mg/L)	2.9 (1.2 –6.7)	3.1 (1.2 –7.5)	2.7 (1.0 –6.5)	0.125
Anemia, %	133 (28.5)	64 (32.5%)	69 (25.6%)	0.073
LVEF (%)	41.6 (14.4)	39.8 (13.8)	44.0 (14.9)	< 0.001
Pulmonary hypertension, %	117 (25.1%)	65 (33.2%)	52 (19.3%)	0.001
*Tricuspid regurgitation, %*				0.004
No	43 (9.2%)	17 (8.7%)	26 (9.6%)	
Mild	197 (42.3%)	70 (35.7%)	127 (47.0%)	
Moderate	139 (29.8%)	57 (29.1%)	82 (30.4%)	
Severe	87 (18.7%)	52 (26.5%)	35 (13.0%)	
RVDd (mm)	39.8 (8.21)	40.3 (8.94)	39.4 (7.63)	
Body mass index (kg/M2)	24.2 (5.7)	23.6 (5.7)	24.6 (5.8)	0.068
Antisterone, %	415 (89.1%)	177 (90.3%)	238 (88.1%)	0.451
ACEI/ARB, %	365 (78.3%)	151 (77.0%)	214 (79.3%)	0.566
Beta-blocker, %	373 (80.0%)	151 (77.0%)	222 (82.2%)	0.167
Aspirin, %	198 (42.5%)	85 (43.4%)	113 (41.9%)	0.744
MELD	8.5 (7.2–10.9)	8.9 (7.4–11.9)	8.3 (7.1–10.2)	0.004
MELD_XI	11.3 (9.4–13.8)	11.8 (9.4–15.2)	11.0 (9.4–13.2)	0.008
MELD_sodium	8.6 (6.5–12.4)	9.4 (6.9–14.0)	8.3 (6.3–11.0)	0.004
MELD_albumin	12.3 (9.6–15.2)	13.1 (10.6–16.4)	11.6 (9.0–14.6)	< 0.001

Data are presented as mean (SD) or median (interquartile range), or n (%). *proBNP* N-terminal pro-brain natriuretic peptide, *NYHA* New York Heart Association functional, *ALT* alanine aminotransferase, *AST* aspartate aminotransaminase, *LVEF* left ventricular ejection fraction, *RVDd* right ventricular diastolic diameter, *ACEI* angiotensin converting enzyme inhibitors, *ARB* angiotensin receptor blocker

### Agreement of measurements

**Fig. 3:**
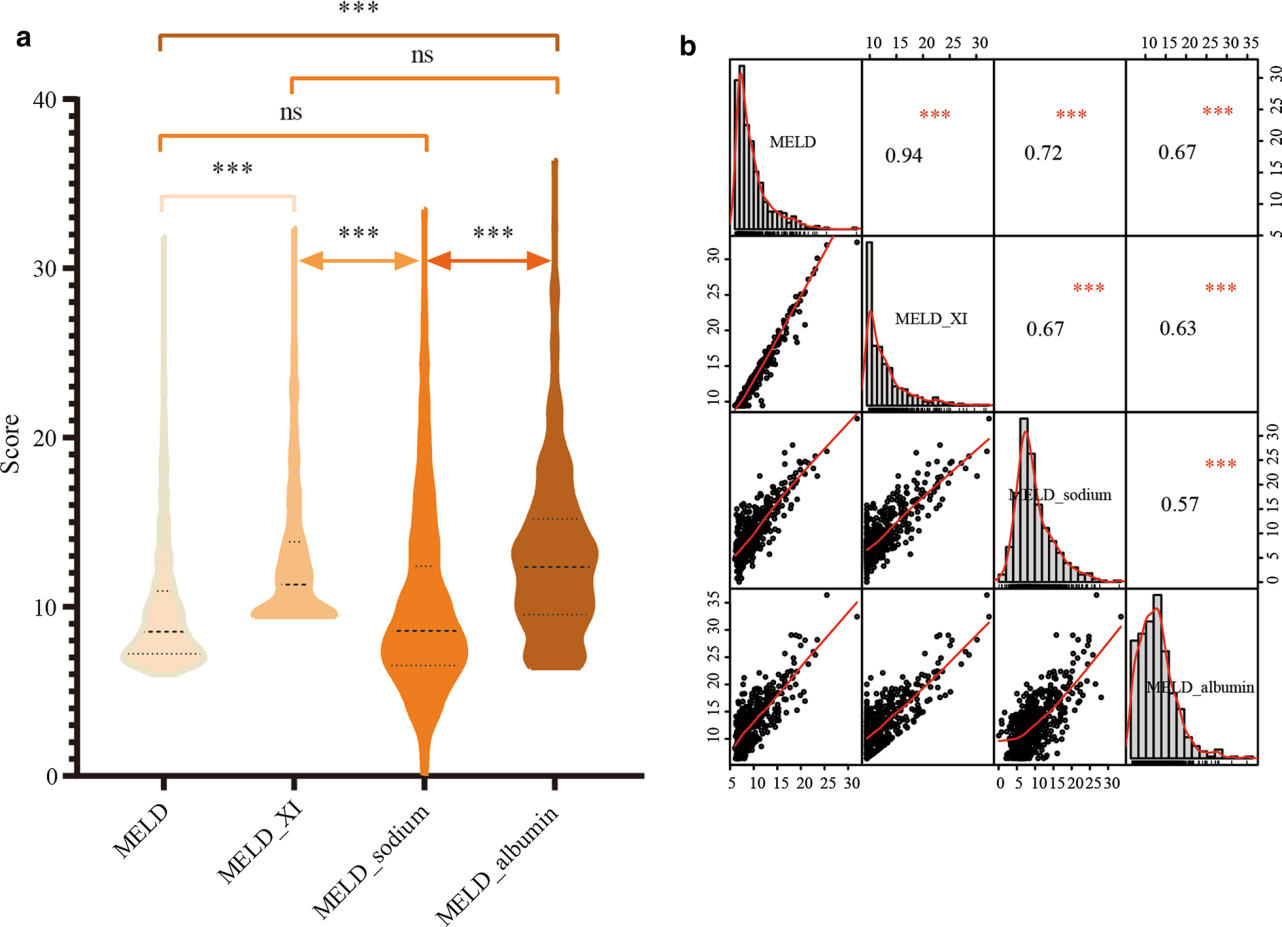
Quantitative distribution of the model for end-stage liver disease (MELD) score and its 3 modifications (**a**). Pairwise Spearman correlations among MELD score and its 3 modifications (**b**). ***, *P* < 0.001

Figure 3a presents the estimates of the MELD score and its 3 modifications using the four formulas. The median (interquartile) values of the MELD, MELD_XI, MELD_sodium and MELD_albumin scores were 7.2 (8.5–10.9), 11.3 (9.4–13.8), 8.6 (6.5–12.4) and 12.3 (9.6–15.2), respectively. The MELD_XI and MELD_albumin scores were significantly higher than the MELD score (all *P* value < 0.001), and the MELD_sodium score was similar to the MELD score. Pairwise Spearman correlation test was performed among the MELD score and its 3 modifications. MELD correlated better with MELD_XI (r value = 0.94, *P* < 0.001) than the 2 other modified MELD scores (r value for MELD_sodium = 0.72, *P* < 0.001; r value for MELD_albumin = 0.67, *P* < 0.001) (Fig. 3b).

### Impact of MELD score and its 3 modifications on mortality

Table 2 shows the main predictors of all-cause mortality by univariate Cox regression analysis. Baseline age, sex, NYHA class, LVEF, proBNP, systolic blood pressure, diastolic blood pressure and pulmonary hypertension. (*P* values < 0.05) were considered potential risk factors for long-term all-cause mortality in patients with AHF. After further adjusting all risk factors at baseline, standardized hazard ratios (HRs) with 95% confidence intervals (CIs) expressing the risk of all-cause mortality were 1.22 (1.06–1.40), 1.20 (1.04–1.39), 1.23 (1.06–1.42) and 1.21 (1.05–1.41) for the MELD, MELD_ XI, MELD_sodium and MELD_albumin scores, respectively, and the corresponding hazard ratios for cardiovascular mortality were 1.30 (1.12–1.50), 1.28 (1.10–1.49), 1.34 (1.14–1.57) and 1.27 (1.07–1.49), respectively (Table 3).

**Table 2:** Univariate analysis of predictors for all-cause mortality

	HR (95% CI)	*P* value
Age (per year increase)	1.00 (1.00, 1.01)	0.016
Male	0.55 (0.41, 0.73)	< 0.001
Diabetes	1.01 (0.74, 1.39)	0.936
Hypertension	0.87 (0.66, 1.15)	0.327
Ischemic heart failure	1.12 (0.82, 1.54)	0.483
*NYHA*		
II	1.00	
III	1.63 (1.05, 2.54)	0.031
IV	2.01 (1.25, 3.21)	0.004
Heart rate (per beats/min increase)	1.00 (0.99, 1.00)	0.521
Systolic blood pressure (per mmHg increase)	0.99 (0.99, 1.00)	0.023
Diastolic blood pressure (per mmHg increase)	0.98 (0.97, 0.99)	0.001
Potassium (per mmol/L increase)	1.08 (0.81, 1.42)	0.612
Uric acid (per mg/dL increase)	1.05 (0.99, 1.10)	0.057
ALT (per U/L increase)	1.00 (1.00, 1.00)	0.598
AST (per U/L increase)	1.00 (1.00, 1.00)	0.659
proBNP (per ng/L increase)	1.00 (1.00, 1.00)	< 0.001
CRP (per mg/L increase)	1.01 (0.99, 1.03)	0.082
Anemia	1.22 (0.82, 1.80)	0.325
LVEF (per 1 unit increase)	0.98 (0.97, 0.99)	0.001
Pulmonary hypertension	1.69 (1.26, 2.28)	0.001
*Tricuspid regurgitation*		
No	1.00	
Mild	0.96 (0.54, 1.71)	0.890
Moderate	1.16 (0.65, 2.09)	0.619
Severe	1.74 (0.96, 3.14)	0.068
RVDd (per mm increase)	1.02 (1.00, 1.04)	0.056
Body mass index (per kg/M2 increase)	0.98 (0.95, 1.00)	0.082
Antisterone	1.13 (0.70, 1.81)	0.622
ACEI/ARB	0.94 (0.67, 1.31)	0.699
Beta-blocker	0.79 (0.57, 1.10)	0.163
Aspirin	1.08 (0.82, 1.44)	0.583

*NYHA* New York Heart Association functional class, *ALT* alanine aminotransferase, *AST* aspartate aminotransaminase, *LVEF* left ventricular ejection fraction, *ACEI* angiotensin converting enzyme inhibitors, *ARB* angiotensin receptor blocker

**Table 3:** Standardized hazard ratios (95% confidence intervals) of mortality

	Unadjusted		Model 1		Model 2	
	HR(95%CI)	*P* value	HR (95%CI)	*P* value	HR (95%CI)	*P* value
*All cause mortality (196/466, 42.1%)*						
MELD	1.23(1.10–1.38)	< 0.001	1.31 (1.16–1.47)	< 0.001	1.21 (1.05–1.40)	0.008
MELD_XI	1.21(1.08–1.36)	0.002	1.28 (1.14–1.45)	< 0.001	1.20 (1.04–1.39)	0.014
MELD_sodium	1.29(1.14–1.46)	< 0.001	1.36 (1.19–1.54)	< 0.001	1.23 (1.06–1.43)	0.005
MELD_albumin	1.27(1.13–1.42)	< 0.001	1.31 (1.16–1.47)	< 0.001	1.22 (1.05–1.41)	0.012
*Cardiovascular mortality (158/466, 33.9%)*						
MELD	1.31(1.17–1.48)	< 0.001	1.39 (1.23–1.56)	< 0.001	1.29 (1.11–1.50)	0.001
MELD_XI	1.29(1.14–1.46)	< 0.001	1.36 (1.20–1.53)	< 0.001	1.27 (1.09–1.48)	0.002
MELD_sodium	1.41(1.23–1.60)	< 0.001	1.48 (1.29–1.69)	< 0.001	1.34 (1.14–1.56)	< 0.001
MELD_albumin	1.31(1.15–1.48)	< 0.001	1.35 (1.18–1.53)	< 0.001	1.27 (1.07–1.50)	0.006

Model 1 was adjusted for age and sex

Model2 was adjusted age, sex, NYHA class, LVEF, proBNP, systolic blood pressure, diastolic blood pressure and pulmonary hypertension

### Prognostic accuracy

**Fig. 4:**
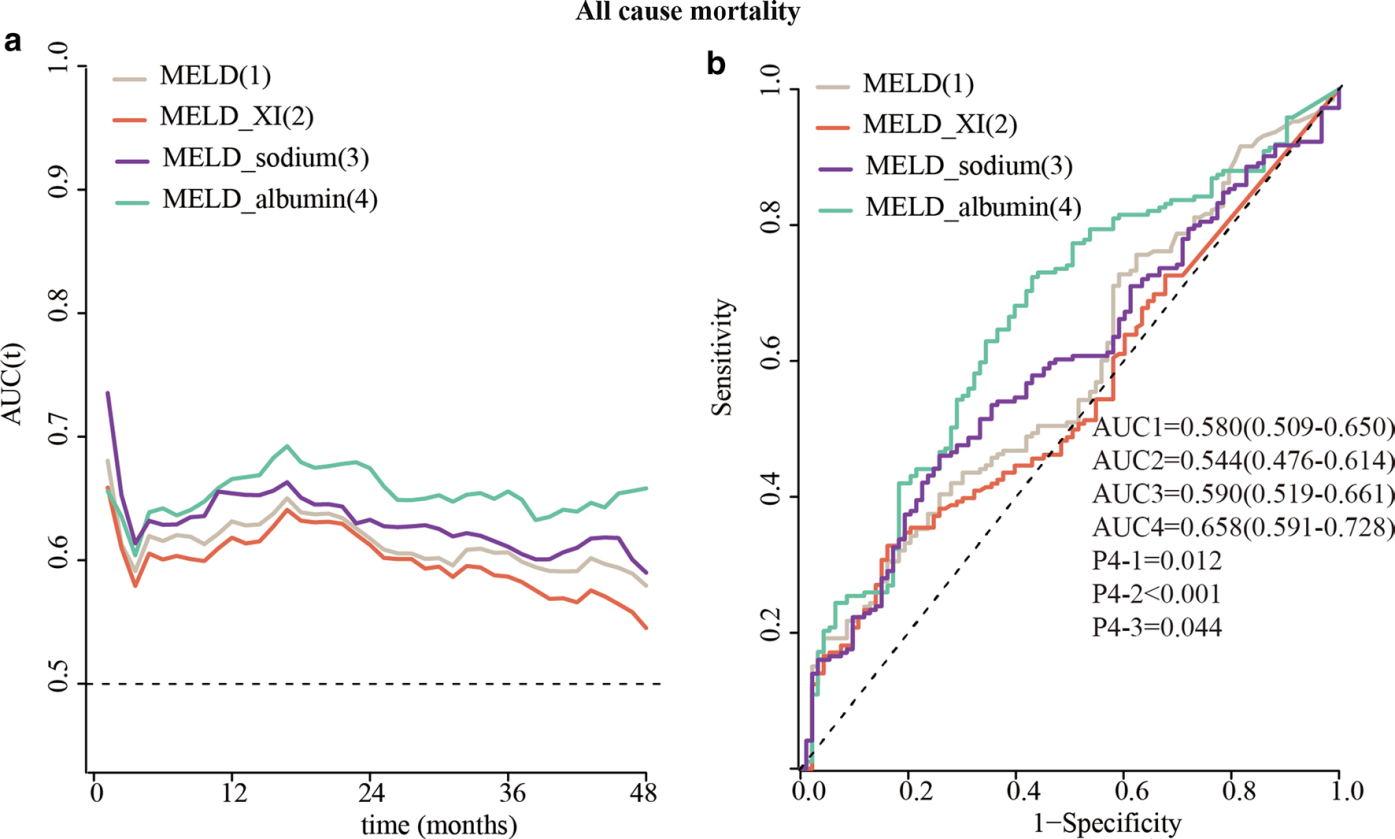
Time-dependent ROC analysis (**a**) and areas under the curve (**b**) of the model for end-stage liver disease (MELD) score and its 3 modifications for all-cause mortality

**Fig. 5:**
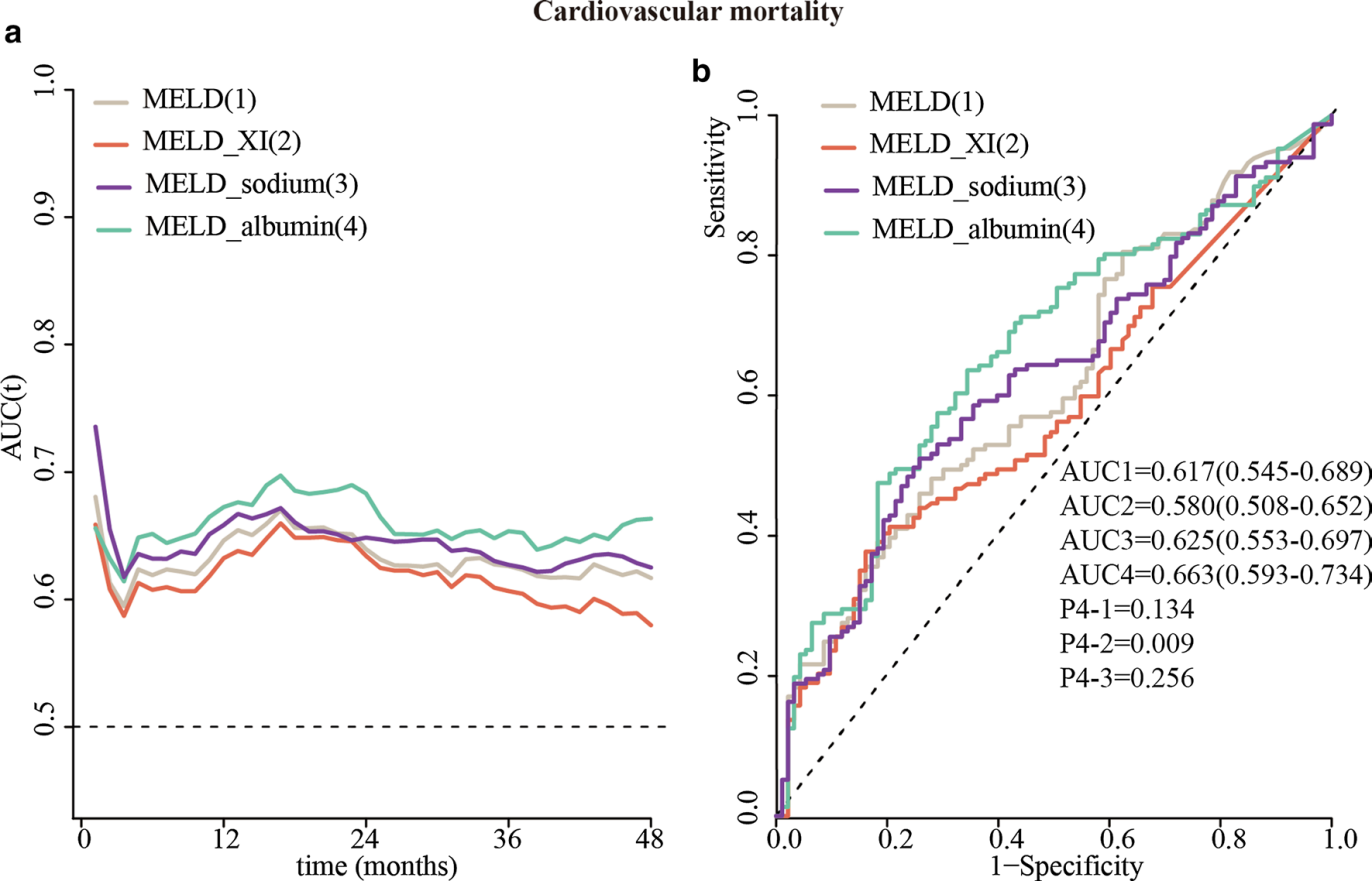
Time-dependent ROC analysis (**a**) and areas under the curve (**b**) of the model for end-stage liver disease (MELD) score and its 3 modifications for cardiovascular mortality

The predictive accuracy of the MELD, MELD_XI, MELD_sodium and MELD_albumin scores was compared using time-dependent ROC curves. If we considered the entire follow-up period, the MELD_albumin score showed the best prognostic accuracy for all-cause and cardiovascular mortality in nearly the entire follow-up period (Figs. 4a, 5a). The quantified AUCs with a four-year cut-off for all-cause mortality outcome were 0.580 (0.509–0.650) for the MELD score, 0.544 (0.476–0.614) for the MELD_XI score, 0.590 (0.519–0.661) for the MELD_sodium score, and 0.658 (0.591–0.728) for the MELD_albumin score. The MELD_albumin score was significantly better than the MELD and MELD_XI for predicting 4-year all-cause mortality risk (all *P* value < 0.016, Fig. 4b).

The quantified AUCs with a four-year cut-off for cardiovascular mortality outcome were 0.617 (0.545–0.689) for the MELD score, 0.580 (0.508–0.652) for the MELD_XI score, 0.625 (0.553–0.697) for the MELD_sodium score, and 0.663 (0.593–0.734) for the MELD_albumin score. The MELD_albumin score was significantly better than the MELD_XI score for predicting the 4-year risk of cardiovascular mortality (*P* value = 0.009, Fig. 5b). However, MELD_albumin showed no significant better than the MELD and MELD_sodium score in predicting 4-year cardiovascular mortality risk.

These findings were evaluated by the NRI analysis (Table 4). MELD_albumin score increased the NRI over and beyond MELD (13.1%, *P* = 0.003), MELD_XI (14.8%, *P* = 0.006), and MELD_sodium (11.9%, *P* = 0.006) scores for all-cause mortality. However, MELD_albumin did not increase NRI in the reclassification of cardiovascular mortality.

**Table 4:** Net reclassification improvement by comparing different MELD equation

	All-cause mortality			Cardiovascular mortality		
	NRI (%)	CI (95%)	*P*	NRI (%)	CI (95%)	*P*
MELD_albumin vs MELD	13.1	5.2 to 22.8	0.003	2.5	-8.9 to 9.9	0.595
MELD_albumin vs MELD_XI	14.8	5.5 to 21.6	0.002	3.1	-8.3 to 11.9	0.539
MELD_albumin vs MELD_sodium	11.9	4.6 to 21.9	0.006	3.6	-9.0 to 14.9	0.547

*NRI* net reclassification improvement, *CI* confidence interval

## Discussion

Our study demonstrated that liver and renal function evaluated by the MELD_albumin score could serve as an important predictor of the prognosis of AHF. Our study also indicates for the first time that evaluating liver and renal function using the MELD_albumin score outperforms evaluation using MELD and other modified MELD scores to discriminate the risk of all-cause mortality in AHF patients. The present data extend the usefulness of modified MELD scores to predict clinical events in patients with AHF.

Our findings are consistent with previous studies reporting the prognostic role of liver and renal function on mortality in patients with AHF [3, 6, 7] and add interesting information to the available literature on this topic. The MELD score and its modifications, which have been widely used as prognostic predictors in patients with liver diseases [10, 12], have recently been shown to be associated with worse clinical outcomes in patients with AHF [3, 6, 7]. In the study conducted by Kim et al. [6], elevated MELD, MELD_XI, and MELD_sodium scores were associated with an increased risk for composite endpoint of death, heart transplantation and ventricular-assist device requirement in ambulatory patients with heart failure (HR was 1.10 (1.06–1.14) for MELD; HR was 1.13 (1.07–1.19) for MELD_XI; HR was 1.10 (1.06–1.14) for MELD_sodium). Another study by Biegus et al. [3] demonstrated that the MELD or MELD_XI score at baseline was predictive of 1-year all-cause mortality in patients with AHF (HR was 1.08 (1.02–1.35) for MELD, HR 1.11 (1.05–1.2) for MELD_XI). Similar results were also found in another study by Biegus et al. [7], in which the MELD-XI score was significantly associated with all-cause death (HR 1.11 (1.04–1.17)). Notably, the MELD score has been shown to identify risk in patients supported with a left ventricular assist device [16] and patients who underwent cardiac surgery [17].

Compared to the previous studies, the difference in hazard ratios of our study may arise from the relatively larger sample size, the longer follow-up duration, the value of the MELD score or its modifications, the definition of the outcome and the cofounding factors we adjusted. Notably, our study not only highlighted the prognostic role of the modified MELD score but also indicated that the MELD_albumin score was superior to the MELD and other modified MELD scores in predicting the risk of mortality. Our study supports the incorporation of serum albumin into the modified MELD score to provide additional risk information in patients with AHF.

The underlying mechanism is that venous congestion and hypoperfusion can contribute to elevated liver enzymes and creatinine, resulting in cardio–renal–hepatic syndrome [18]. The progression of liver dysfunction in patients with AHF is complex and may follow any of several pathways. Biochemical markers of cholestasis, such as γ-glutamyl transpeptidase, bilirubin, and alkaline phosphatase, are increased in the plasma of AHF patients with elevated central venous pressure [19]. Hypoperfusion-induced hypoxic liver injury is also the major cause of massively elevated aminotransferase levels in patients with AHF, and the elevation of these levels is a risk factor for mortality [20]. In the setting of renal dysfunction, congestion was a stronger predictor of worsening renal function than cardiac output or mean arterial pressure in patients with AHF [21, 22]. Congestion is the main profile of diastolic dysfunction. Interestingly, a study also indicated that renal insufficiency is more important in determining the risk of mortality in patients with diastolic cardiac dysfunction than in those with systolic dysfunction [23]. The increase in central venous pressure is associated with worsen renal function through several mechanisms including the decrease in renal blood flow and interstitial fibrosis [24].

There are several limitations in our study. First, the empirically chosen Chinese population was a major limitation. Validation in other population should be performed to verify our results. Second, although we included a set of confounding factors and indicated the strong prognostic power of the MELD score and its modifications in patients with AHF, unmeasured cofounding factors may also play a role. Finally, the MELD score or its modifications are subject to laboratory variations, and previous oral medications such as diuretics can impact sodium and creatinine excretion, which could confound the results.

## Conclusions

Quantifying liver and renal function using the MELD_albumin score in patients with acute heart failure assesses the risk of mortality more accurately than the commonly used MELD score or its other modifications.

## Data Availability

The datasets used and/or analyzed during the current study are available from the corresponding author on reasonable request.

## Abbreviations

AHF: Acute heart failure
ALT: Alanine aminotransferase
AST: Aspartate aminotransaminase
AUC: Areas under the curve
BMI: Body mass index
CI: Confidence interval
HF: Heart failure
HR: Hazard ratio
INR: International normalized ratio
LVEF: Left ventricular ejection fraction
MELD: Model for end-stage liver disease
MELD_XI: MELD excluding the international normalized ratio
MELD_sodium: MELD including sodium
NRI: Net reclassification improvement
NYHA: New York Heart Association
proBNP: Pro-brain natriuretic peptide
ROC: Receiver operating characteristic
